# Advancing in Cesium Retention: Application of Magnesium Phosphate Cement Composites

**DOI:** 10.3390/ma17092132

**Published:** 2024-05-01

**Authors:** Sana Gharsallah, Nawel Khitouni, Abdulrahman Mallah, Abdulrahman Alsawi, Abdullah H. Alluhayb, Mohamed Khitouni, Clarence Charnay, Mahmoud Chemingui

**Affiliations:** 1 Laboratory of Inorganic Chemistry, LR17-ES-07, Faculty of Science, University of Sfax, Sfax 3018, Tunisia; sana.gharsallah.etud@fss.usf.tn (S.G.); khitouninawel@yahoo.fr (N.K.); mahmoud.chmingui@fss.usf.tn (M.C.); 2 Department of Chemistry, College of Science, Qassim University, Buraydah 51452, Saudi Arabia; ah.alluhayb@qu.edu.sa (A.H.A.); kh.mohamed@qu.edu.sa (M.K.); 3 Department of Physics, College of Science, Qassim University, Buraydah 51452, Saudi Arabia; ansaoy@qu.edu.sa; 4 Charles Gerhard Institut, UMR-5253 CNRS-UM-ENSCM, University of Montpellier, Place E, Bataillon, CEDEX 5, F-34095 Montpellier, France; clarence.charnay@umontpellier.fr

**Keywords:** magnesium phosphate cement (MPC), X-ray diffraction, scanning electron microscope, adsorption, cesium

## Abstract

A serious risk that harms the safe use of water and affects aquatic ecosystems is water pollution. This occurs when the water’s natural equilibrium is disrupted by an excessive amount of substances, both naturally occurring and as a byproduct of human activities, that have varied degrees of toxicity. Radiation from Cs isotopes, which are common components of radioactive waste and are known for their long half-lives (30 years), which are longer than the natural decay processes, is a major source of contamination. Adsorption is a commonly used technique for reducing this kind of contamination, and zeolite chabazite has been chosen as the best adsorbent for cesium in this particular situation. The purpose of this research is to investigate a composite material based on magnesium phosphate cement (MPC). Magnesium oxide (MgO), potassium dihydrogen phosphate (KH_2_PO_4_), and properly selected retarders are used to create the MPC. The optimal conditions for this composite material are investigated through the utilization of X-ray diffraction, scanning electron microscopy, BET surface area analysis, and atomic absorption spectroscopy. The principal aim is to enable innovations in the elimination of radioactive waste-contaminated water using effective cesium removal. The most promising results were obtained by using KH_2_PO_4_ as an acid, and MgO as a base, and aiming for an M/P ratio of two or four. Furthermore, we chose zeolite chabazite as a crucial component. The best adsorption abilities for Cs were found at Q_ads_ = 106.997 mg/g for S_2_ and Q_ads_ = 122.108 mg/g for S_1_. As a result, zeolite is an eco-friendly material that is a potential usage option, with many benefits, such as low prices, stability, and ease of regeneration and use.

## 1. Introduction

The intriguing world of magnesium phosphate cement (MPC) has surged into prominence in recent years, propelled by its remarkable attributes and versatile utility across various domains. Composed predominantly of magnesium oxide (MgO) and a phosphate solution, typically derived from sources such as ammonium dihydrogen phosphate ((NH_4_)_2_HPO_4_) or potassium dihydrogen phosphate (KH_2_PO_4_), MPC undergoes a meticulously orchestrated chemical transformation upon blending these constituents in precise proportions. This intricate alchemy results in the emergence of magnesium phosphate crystals, acting as a potent binder, thus laying the foundation for a host of construction and engineering applications [1,2].

Central to MPC’s formulation is the convergence of magnesium, phosphate, and ammonium ions, a process that culminates in the precipitation of the phosphate mineral struvite (MgNH_4_PO_4_·6H_2_O) under conducive environmental conditions [3]. Moreover, the synthesis of a modified struvite variant, known as K-struvite, introduces potassium ions to partially substitute magnesium ions, yielding the potassium magnesium ammonium phosphate complex (KMgPO_4_·6H_2_O) [4]. This nuanced variation extends the repertoire of MPC, offering enhanced properties and broader functionality in diverse contexts.

Beyond its chemical intricacies, MPC embodies a promising avenue for sustainable construction practices, owing to its environmentally friendly composition and reduced carbon footprint compared to traditional cementitious materials. Furthermore, its rapid setting characteristics and excellent mechanical properties make it an attractive option for infrastructure projects where time and durability are paramount considerations [3,4]. 

In essence, the evolution of magnesium phosphate cement epitomizes the convergence of scientific ingenuity and practical innovation, unlocking new possibilities for resilient and eco-conscious construction methodologies in the modern era.

With its distinctive composition and properties, magnesium phosphate cement (MPC) emerges as a compelling alternative to conventional Portland cement, showcasing both practical utility and environmental sustainability. Notably, MPC exhibits rapid setting kinetics and early stage strength development, positioning it as an optimal choice for applications requiring expedited construction or maintenance interventions [5]. This accelerated setting time is attributed to the formation of robust hydration products, including struvite and K-struvite crystals, which contribute to the material’s rapid strength gain [6,7].

Furthermore, MPC demonstrates commendable compressive strength comparable to that of Portland cement, ensuring structural robustness across diverse engineering contexts [8,9]. Its notable resistance to chemical degradation stands as a critical attribute, mitigating the impact of environmental factors such as acid attack, sulfate exposure, and chloride ingress, thereby prolonging material longevity [10,11,12]. Additionally, the observed reduction in contraction during the curing phase contributes significantly to enhanced structural durability, mitigating crack formation and fortifying long-term performance [10,11].

Moreover, MPC’s exceptional fire resistance underscores its suitability for applications subjected to elevated temperatures, surpassing the performance of conventional cementitious materials under fire exposure [13]. Beyond its mechanical and chemical properties, MPC exhibits low shrinkage characteristics, contributing to reduced internal stresses and enhanced durability [14]. This multifaceted performance profile positions MPC as a versatile and resilient material suitable for a wide range of engineering applications, including nuclear waste encapsulation, maritime infrastructure development, and comprehensive infrastructure rehabilitation initiatives. As research in this field continues to advance, MPC holds promise as a transformative solution in various academic and industrial settings, offering enduring performance and sustainability across a broad spectrum of applications [6,15].

Zeolite chabazite, a tectosilicate mineral, is characterized by its large specific surface area and remarkable porosity, making it one of the most notable natural zeolites. Chabazite’s structural elements include a six-membered double prismatic pyramid that is softly joined by four rings to form tight, ordered cubes [7]. This small-pore zeolite is an excellent ion exchanger, which enables it to effectively remove contaminants from a variety of effluents. Chabazite’s physicochemical characteristics, particularly its sorption and cation exchange capacities, further increase its usefulness. Because of its ease of use, high yield, exceptional chemical stability, cost-effectiveness, specific cation selectivity, and thermal resilience, the zeolites-based adsorption technology is very promising [7,12].

In this study, our aim is to unlock the vast potential of magnesium phosphate cement (MPC) by fortifying its matrix with the remarkable zeolite chabazite. With a targeted focus on cesium, our strategic integration seeks not only to enhance the cement’s current attributes but also to explore its potential for significantly improving adsorption capabilities. Through meticulous experimentation and innovative methodologies, we endeavor to propel MPC to new heights of performance, presenting exciting opportunities for advancing environmental remediation and sustainable construction practices.

Water pollution stemming from radioactive waste presents a significant and multifaceted challenge with far-reaching implications for both human health and the environment. The extent of this problem spans across various dimensions, encompassing the contamination of freshwater sources, marine ecosystems, and groundwater reserves. Radioactive pollutants can persist in water bodies for extended periods, posing grave risks to aquatic life and biodiversity. Furthermore, the release of radioactive contaminants into drinking water supplies threatens human health, potentially leading to long-term health effects such as cancer, genetic mutations, and organ damage. Additionally, the environmental consequences of water pollution from radioactive waste are profound, with ecosystems experiencing disruptions in nutrient cycling, species decline, and habitat degradation. The cumulative impact of these factors underscores the urgency of addressing this issue through rigorous regulatory measures, effective waste management strategies, and proactive mitigation efforts to safeguard both human well-being and ecological integrity.

In the realm of radioactive materials, cesium-137 isotopes stand out as primary contaminants within radioactive waste streams [12,13]. Their significant radiotoxicity stems from an extended half-life of approximately 30 years, a duration that far exceeds the timeframe for natural decay to adequately diminish their impact [14,15]. Of particular concern is the challenge posed by cesium-137′s propensity to exist in a soluble salt state, rendering it highly water-soluble and thus more prone to dispersion in the environment.

Originating from nuclear processes, cesium-137 exhibits a profound level of radiotoxicity, making it particularly hazardous to human health. Its ability to permeate bodily tissues freely allows it to accumulate predominantly in muscle tissue, amplifying its deleterious effects [16,17]. Furthermore, cesium-137’s impact extends beyond immediate tissue damage; it can also induce detrimental effects on vital organs such as the liver and kidneys, while exacerbating susceptibility to cardiovascular diseases [16,17].

Given these formidable characteristics, the effective management and remediation of cesium-137 contamination represent paramount challenges in the field of environmental and public health. Understanding the complex interplay between cesium-137 and its surrounding environment is crucial for developing robust strategies for containment, remediation, and ultimately mitigating its adverse impacts on both human health and the environment [1,17].

In the field of cesium (Cs) adsorption, a multitude of materials have emerged as promising candidates for effectively removing Cs ions from aqueous solutions. Among these, zeolites stand out for their crystalline structure, high surface area, and strong ion-exchange properties. Notably, zeolites like clinoptilolite and chabazite exhibit exceptional affinity for Cs ions, making them valuable tools in environmental remediation efforts.

Our study delves into the development of a composite material with magnesium phosphate cement at its core, aiming to enhance Cs adsorption efficiency. Through the integration of zeolite chabazite as a reinforcing agent, we’ve pioneered a novel composite material poised to revolutionize environmental remediation and radioactive waste management. This composite material showcases superior adsorption capabilities while effectively minimizing the dispersal of Cs pollutants. Our findings highlight the promising potential of this innovative solution in tackling critical environmental challenges.

## 2. Materials and Methods

### 2.1. Materials

MPC pastes were produced by blending a powder mixture of potassium dihydrogen phosphate (KDP > 99%, sourced from SIGMA-ALDRICH, Darmstadt, Germany), high-purity magnesia (MgO > 99%, obtained from Merck, Darmstadt, Germany), and zeolite of the chabazite type (Zeolite Zn-324-09 BOWIE chabazite, Azlb-Ca) known as Zeofume (dv50-15 micron). To regulate the paste’s setting time, borax was employed as a set retarder.

The magnesia underwent a meticulous calcination process lasting two hours at a temperature of 1005 °C, strategically employed to refine its properties by reducing its reactivity. Noteworthy is the deliberate exclusion of aggregates from the experimental setup to prevent potential interference from associated contaminants. The synthesis protocol commenced with the fusion of acid and magnesium, supplemented by the introduction of borax as a retardant agent. Employing two distinct ratios, Mg/P = 2 and Mg/P = 4, precise measurements of solvent levels were meticulously observed. Subsequently, the introduction of zeolite into the mixture marked a pivotal stage in the synthesis process.

### 2.2. Analysis Method

To evaluate the characteristics of the MPC pastes, a phase characterization was conducted. Powder X-ray diffraction (XRD) analysis (Philips, Farnborough, UK) was performed using an X’per PRO PANalytical equipment with CuKα radiation (λcu = 1.54 Å) to identify the crystalline phases present in the MPC pastes. Scanning electron microscopy (SEM) (SEM, (JEOL)-Japon, Tokyo, Japan) was employed to investigate the microstructure of the cement. Additionally, the Brunauer-Emmett-Teller (BET) method using the TriStar 3000 V6.06 A (Norcross, GA, USA)was utilized to determine the specific surface area of the cement. Furthermore, atomic flame absorption spectroscopy (Waltham, MA, USA)was employed to measure the adsorption rate of Cs.

### 2.3. Parameter Optimization

The chemical reaction between an acidic and an alkaline component produces magnesium phosphate cement, and an acid-base type cement that neutralizes rapidly and has a neutral pH due to several factors that can affect the structure. We tried to optimize several cement-influencing factors in our investigation. We examined the usage of setting retarders, the Mg/P ratio, the amount of additional water, and various bases and acids among these parameters.

#### 2.3.1. Base

A variety of bases, including ZnO, CaO, MgO, and Mg(OH)_2_, were employed. These bases were either employed uncalcined or calcined at varying temperatures. We can deduct from these initial experiments that MgO is the ideal base to utilize and that calcination significantly alters MgO’s reactivity. Table 1 presents different results of the application of MgO.

#### 2.3.2. Acid

Following base selection, experiments were conducted to determine the optimal acid source for this investigation. A variety of acids, including powdered or crystallized KH_2_PO_4_, K_2_HPO_4_, H_3_PO_4_, MgHPO_4_·3H_2_O, and NH_4_H_2_PO_4_, were investigated. Table 2 arranges the formulations into groups. We selected the optimal acid KH_2_PO_4_ (powder) for the remaining work after these tests.

#### 2.3.3. Mg/P Ratio

The evolution of microstructural characteristics is significantly influenced by the molar ratio of magnesia to phosphate, or Mg/P. Following the selection of the optimal ${PO}_{4}^{3-}$ and base source, the following molar ratios of Mg/P were investigated: 1, 2, 3, 4, 5, 8, 12. Table 3 presents a summary of the results. The setting time is significantly influenced by the Mg/P ratio; the higher the ratio, the shorter the setting time, and the weaker and more brittle the material becomes. We use MgO, KH_2_PO_4_, and a Mg/P ratio of 2 or 4 for the remaining work.

#### 2.3.4. Setting Retarders

The setting time emerges as a pivotal parameter in cement synthesis, owing to its direct association with the reaction kinetics governing the interaction between phosphate and magnesia. Primarily, this temporal characteristic is dictated by the presence of phosphate molecules introduced into the solution through Mg^2+^ ions. Furthermore, the calcination process of MgO, leading to reduced reactivity, exerts a pronounced influence on the setting time. Moreover, variations in the ratios of cement to water and the Mg/P ratio significantly modulate this parameter. Notably, cement setting can occur rapidly, even instantaneously under specific conditions, necessitating the implementation of setting retarders to enhance handling. Various retarders are utilized, with their respective effects detailed in Table 4.

The indispensability of setting retarders in the reaction process is underscored by experiments conducted without their presence, resulting in instantaneous setting, compromised handling, and the inability to achieve homogeneity in paste formation. Optimal outcomes are achieved through the utilization of boron derivatives. Given the comparable impact of borax and boric acid on the setting process, borax was selected as the preferred setting retarder for subsequent experimental procedures.

#### 2.3.5. Quantity of Water

The quantity of water provided is one of the basic factors that affect the dose of the cement mixture. Because the water mass ratio (*w*/*c*) creates porosity in the material, it affects the qualities of hardened cement, especially strength and durability. When a lot of water is added, we observe that a homogenous cement paste does not form, the setting process moves slowly, and the structure becomes fragile thanks to the formation of porosity. The quantity of hydrates generated decreases with less water added, which lowers the ultimate strengths. For hydration to be complete, it is sufficient to add enough water (have an optimal quantity of water). Table 5 presents the results of varying the quantity of water added to determine its impact on the structure. Setting retarders is not added during the preparation of the various cement pastes. The pH range of all synthesized materials was 8 to 9.

In conclusion, through meticulous adjustments of various parameters, our findings underscore the superiority of calcined MgO as the optimal base material, KH_2_PO_4_ as an effective acid component, and a Mg/P ratio of 2 or 4 for achieving optimal performance. The selection of borax as a setting retarder was deliberate, with strict adherence to a maximum quantity of 5% in the cement mixture, as our tests revealed the exceptional efficacy of borax within the boric family of compounds. Additionally, the judicious use of water is imperative to ensure the creation of a homogeneous mixture, with careful consideration to avoid excessive moisture that may compromise the structural integrity, resulting in undesired crumbliness. These significant findings highlight the importance of precise parameter selection in optimizing the performance and characteristics of composite materials for various applications.

#### 2.3.6. Preparation

In the synthesis protocol, magnesium and acid are combined with the retarding agent (borax) and the Mg/P ratio (which should be either 2 or 4). Water measurements are carefully followed when blending this mixture, and the pH is checked to make sure it stays within the range for zeolite stability. The specified quantities of the chosen reinforcements, which make up 50% of the cement quantity for the Cs adsorption materials, are next added. Until a homogenous paste is achieved, mixing is continued. All of the materials produced for Cs adsorption are synthesized using this standardized protocol, whose composition is shown in Table 6.

## 3. Results

### 3.1. SEM Analysis

**Figure 1:**
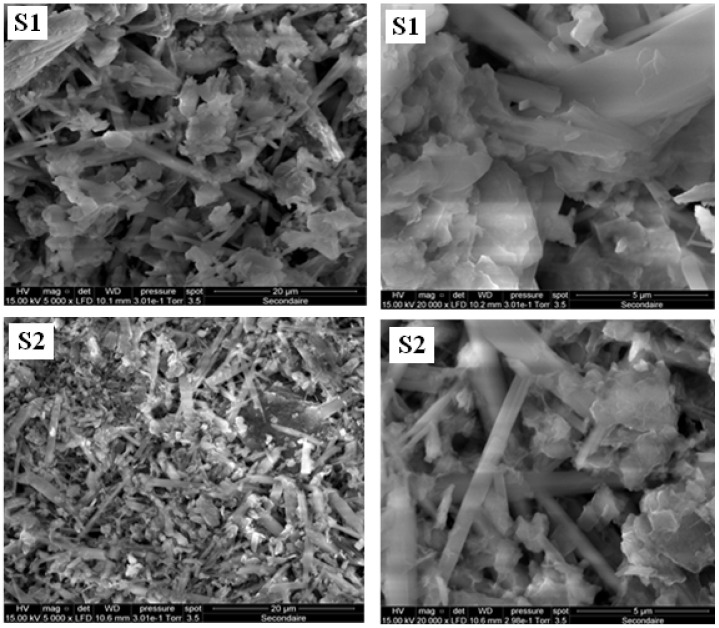
SEM images of the samples S_1_ and S_2_ (low and high magnification).

The scanning electron microscopy (SEM) analysis unveiled intricate microstructures characterized by elongated, stick-like formations for both zeolite-prepared materials (Figure 1). These elongated structures manifest random and tangled arrangements within the micrographs, thus contributing to the material’s inherent compactness. Additionally, an eminent observation entailed a significant degree of porosity exhibited by both samples, attributable to the random overlapping and entanglement of these elongated rods. This phenomenon engenders interconnected void spaces within the material matrix. Such intricate porosity profiles markedly augment the material’s surface area, thereby potentially exerting a profound influence on its overarching characteristics and adsorption efficacy [3,18,19].

### 3.2. BET-Analysis

**Figure 2:**
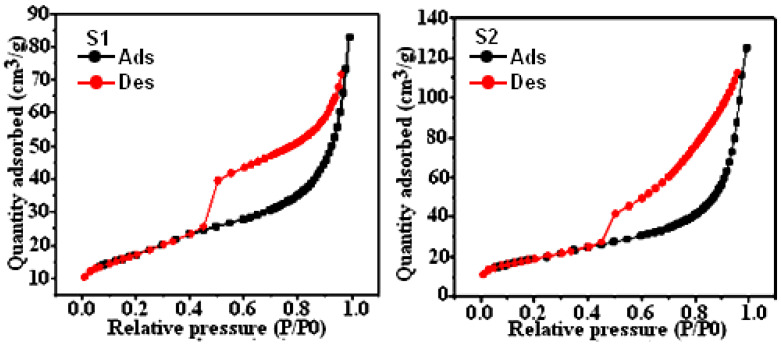
N2 adsorption–desorption curve for composites samples S_1_ and S_2_.

With a pore size of 8 nm and a pore volume of 0.111959 cm^3^/g, composite S_1_ has a BET-specific surface area of 62.9793 m^2^/g (Figure 2). Conversely, composite S_2_ has a slightly larger pore size of 11 nm and a pore volume of 0.181611 cm^3^/g, resulting in a BET-specific surface area of 67.5971 m^2^/g. There is low energy absorption in both composites as indicated by the Type III N2 adsorption/desorption isotherm. Furthermore, both examples exhibit a Type E hysteresis, indicating the presence of bottle-shaped holes based on the IUPAC classification [3,20].

### 3.3. XRD Analysis

**Figure 3:**
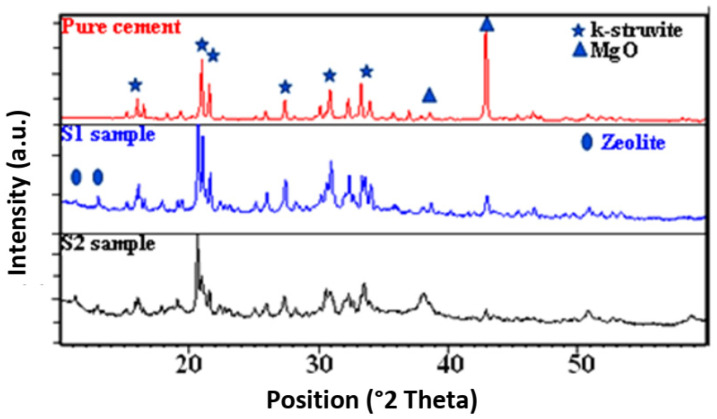
Comparison of X-ray diffraction patterns of pure cement with the samples S_1_ and S_2_ prepared with zeolite.

Upon analyzing the peaks of the X-ray spectrum in both samples (Figure 3), it is evident that K-struvite is present at 2θ = 15°, in the range of 2θ = 20–21°, and the region of 2θ = 31–34°. Furthermore, distinct zeolite peaks were detected between 2θ = 10° and 13°, confirming the structural stability. Moreover, distinctive MgO peaks appeared at 2θ = 37° and 44°, adding validity to the results [3,20,21].

### 3.4. Adsorption

To evaluate their adsorption efficacy, two distinct materials were selected for testing, and in order to obtain precise measurements, 15 tubes of varying concentrations were prepared for each material. The solid utilized had a mass of *m* = 500 mg. For the initial stock solution, a volume of 500 mL with a concentration of *C*_0_ = 25 mM was employed. The calculated mass of CsNO_3_ was found to be 2.436 g. Each tube, with a volume of *V* = 20 mL, underwent preparation before being left to stir overnight. Subsequently, a centrifugation procedure was conducted for five minutes at 500 rpm. Following further dilution, the final dilution was increased by a factor of ten. The adsorbent capacity of these materials was subsequently determined using flame atomic adsorption. Using the following equation [20,22], the amount of cesium sorbed at equilibrium (*q_e_*) (mg/g), was calculated using Equation (1):

(1) $$q_{e}=\frac{\left(C_{0}-C_{e}\right)\times V}{m}$$

where *C*_0_ and *Ce* are the initial and final concentrations of fluoride in solution (mg/L), *V* is the volume of solution (L) and m is the weight of adsorbent (g).

**Figure 4:**
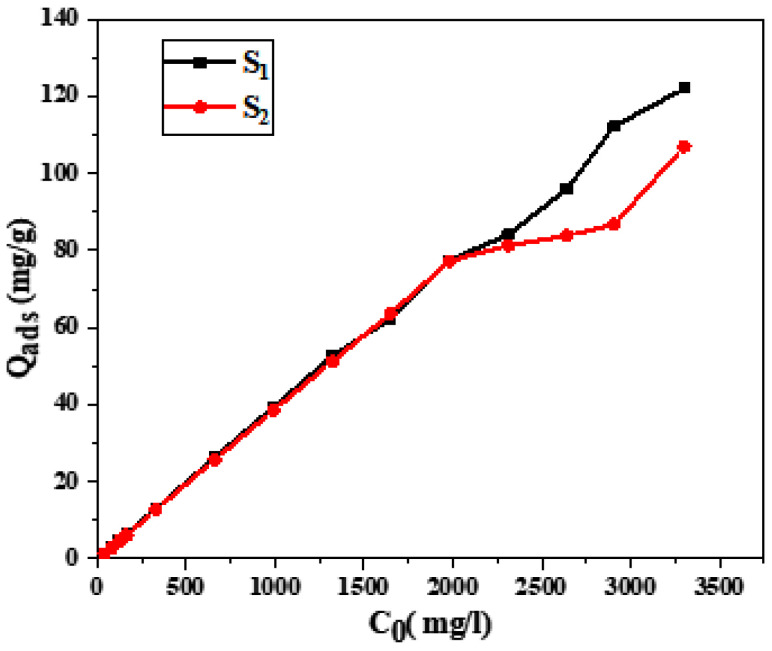
Influence of the initial concentration of cesium on adsorption.

A significant adsorption capacity was found by analyzing the Cs adsorption in sample S_1_, with a maximal quantity recorded at Q_ads_ = 122.108 mg/g (Figure 4). Similarly, sample S_2_ displayed a significant peak adsorbed quantity of 106.997 mg/g. It is essential to remember that adsorption intensifies with concentration. Consequently, the adsorption process benefits by using more zeolite in the mixture.

The study investigated the adsorption behavior of Cs+ ions at two distinct concentration levels using two different adsorbents under identical temperature conditions. To elucidate the adsorption dynamics and unveil the equilibrium relationship between aqueous phase concentration and adsorbent particle concentration, we employed the Langmuir, Freundlich, Temkin, and Dubinin-Radushkevich models. The Langmuir isotherm stands out as a straightforward yet highly effective model for describing adsorption phenomena, particularly when a finite number of identical adsorption sites on the surface are involved, leading to monolayer adsorption. Mathematically, this adsorption process is succinctly captured by the Langmuir equation, which forms a fundamental component of this model [23,24].

The Langmuir equation is expressed using Equation (2):

(2) $$\frac{1}{\;\;\;q_{e}\;}=\frac{1}{q_{m}}+\frac{1}{{bq}_{m}C_{e}}$$

A linear graph with a slope of $\frac{1}{{bq}_{m}}\;$and an intercept of $\frac{1}{q_{m}}$ was produced by the plots of $\frac{1}{\;q_{e}\;}=f(\frac{1}{C_{e}})$, where *b* is the Langmuir adsorption constant (L/mg) and *q_m_* is the maximal adsorption capacity (mg/g).

The Freundlich isotherm model proves versatile in describing sorption data on heterogeneous surfaces, particularly demonstrating efficacy at lower and intermediate concentrations. Unlike the Langmuir isotherm, which assumes monolayer adsorption on homogeneous surfaces, the Freundlich model accommodates the heterogeneous nature of surfaces by allowing for multiple sorption site types. This feature enables a more comprehensive understanding of adsorption behavior, as it acknowledges the varying affinities and interactions between the adsorbate molecules and the surface. Moreover, the Freundlich model’s non-linear relationship between sorbate concentration and adsorption capacity provides insights into the complex mechanisms underlying sorption processes, offering valuable information for optimizing adsorption systems across diverse environmental and industrial contexts [25].

This model can be expressed by Equation (3):

(3) $$\log q_{e}=\log K_{f}+\frac{1}{n}\;\log C_{e}$$

where an approximate measure of the adsorption capacity is given by the constant *K_f_* (mg/g). The degree of surface heterogeneity or the strength of adsorption is indicated by the parameter ‘n’. When log(*q_e_*) is plotted against log (*C_e_*), a linear connection with an intercept at log (*K_f_*) and a slope of 1/*n* is produced.

The Temkin isotherm model integrates a factor to assess the influence of adsorbate-adsorbent interactions on the adsorption mechanism. At its core, this model posits that as the coverage of the adsorbent surface escalates, the heat of adsorption for each molecule within the adsorbed layer decreases linearly. This incorporation of interaction effects provides a nuanced understanding of the adsorption process, shedding light on the dynamic interplay between adsorbate molecules and the surface. By quantifying the variation in heat of adsorption with surface coverage, the Temkin model offers valuable insights into the energetics of adsorption, facilitating the optimization of adsorption systems for diverse applications ranging from environmental remediation to industrial separations [25,26]. This relationship is mathematically represented by the following Formula (4):

(4) $$q_{e}=\frac{RT}{b}\;\ln A+\frac{RT}{b}\;\ln C_{e}$$

where “*T*” is the temperature (K), “*R*” is the gas constant (J/mol K), and “*b*” is the Temkin constant, which denotes the sorption heat (J/mol). “*C_e_*” stands for equilibrium concentration (mg/L), and “*A*” is the Temkin isotherm constant (L/mg).

In contrast to the Langmuir isotherm, the Dubinin–Radushkevich isotherm offers greater adaptability by not relying on the assumptions of a homogeneous surface and constant sorption potential. This flexibility allows the Dubinin–Radushkevich model to effectively capture adsorption phenomena on heterogeneous surfaces and under varying sorption conditions. By accounting for the non-uniformity of surfaces and the fluctuating sorption potentials, this model provides a more realistic representation of adsorption behavior, enabling more accurate predictions and insights into adsorption processes across a wide range of environmental and industrial scenarios (Table 7).

This model efficiently explains adsorption on heterogeneous surfaces with Gaussian energy distribution by highlighting the multi-layered nature of adsorption and the function of Van der Waals forces. It has practical usage in the chemical and physical adsorption of different ions [3,21]. The Dubinin–Radushkevich model is expressed by Equation (5):

(5) $$\mathrm{Ln}q\;\varepsilon =\ln q_{m}-\beta \;\varepsilon^{2}$$

where *β* is the adsorption energy and *ε* denotes the Polanyi potential, which is computed as *ε* = *RT*ln (1 + 1/*Ce*).

The correlation coefficient values obtained from the Freundlich, Langmuir, Temkin, and Dubinin–Radushkevich models (0.946 for S_1_ and 0.906 for S_2_) suggest that the Langmuir model provides the most accurate characterization of the adsorption process, as evidenced by meticulous analysis of the data in Table 7. According to this model, Cs^+^ adsorption onto the adsorbent surface follows a monolayer adsorption mechanism, indicating the presence of reliable and localized adsorption sites. Moreover, it implies that once the adsorbent reaches saturation, the adsorption capacity remains constant, with no discernible interactions between the adsorbed ions.

Notably, the calculated R_L_ values for S_1_ and S_2_ (0.187 and 0.042, respectively) both fall within the range of 0 to 1 (0 < *R_L_* < 1), indicating favorable adsorption conditions. Furthermore, the composite materials employed in this study exhibited superior adsorption capabilities compared to other adsorbents reported in the literature, as evidenced by the data presented in Table 2. This exceptional performance can be attributed to the unique Cs complexation capacity of these materials, which holds promise for the efficient removal of Cs from contaminated water samples. Table 8 offers a comprehensive comparison of the present adsorbent with others.

Our findings reveal the remarkable adsorption capabilities of the developed materials when compared to existing adsorbents in the literature. Table 8 summarizes the comparative analysis, showcasing the impressive adsorption capacities of our synthesized MPC samples, denoted as S_1_ and S_2_. Notably, S_1_, incorporating zeolite chabazite into the MPC matrix, exhibits a remarkable adsorption capacity of 122.108 mg/g, while S_2_ demonstrates a substantial capacity of 106.997 mg/g. These values significantly surpass those of several established adsorbents, emphasizing the efficacy of our approach in enhancing adsorption performance.

## 4. Conclusions

In conclusion, our study was centered on the synthesis and comprehensive characterization of composite materials based on zeolite and magnesium phosphate cement, with a primary objective of achieving high-performance radioactive particle selective capture. Through systematic experimentation, we successfully formulated inorganic composites optimized for cesium adsorption, with formulations featuring KH_2_PO_4_ as an acid, MgO as a base, and M/P ratios of either 2 or 4 yielding the most promising results. Crucially, chabazite zeolite emerged as a pivotal component, boasting advantageous properties such as high selectivity, excellent thermal stability, and remarkable ion exchange capacity. These inherent benefits not only contribute to its efficacy in industrial wastewater treatment applications but also highlight its crucial role as a pivotal component within the composite matrix, enhancing both performance and cost-effectiveness.

SEM analyses revealed porous rod-like structures crucial for efficient adsorption, while BET analyses confirmed the presence of tubular pores and significant specific surface areas. XRD analysis verified the synthesis of K-struvite as the cement-bonding phase, ensuring structural integrity.

Adsorption data obtained via Atomic Absorption Spectroscopy demonstrated enhanced adsorption capacities with increasing cesium concentrations, with the most effective adsorption capabilities observed at Qads = 106.997 mg/g for S_2_ and Qads = 122.108 mg/g for S_1_.

In summary, our study presents novel composite materials with superior adsorption characteristics, offering a significant advancement over previous research efforts. The integration of chabazite zeolite within the composite matrix enhances both performance and cost-effectiveness, paving the way for effective and sustainable solutions in environmental remediation endeavors.

## Figures and Tables

**Table 1:** Choice of MgO base.

	MgO (g)	KH_2_PO_4_ (g)	Borax (g)	Water (mL)
MgO not calcined	1.097	0.830	0.105	2
MgO (550 °C/3 h)	1.064	0.864	0.099	1.5
MgO (1005 °C/2 h)	1.0054	0.8456	0.0965	3.5
MgO (1200 °C/2 h)	1.074	0.844	0.098	1.5
MgO (1500 °C/6 h)	1.0664	0.8128	0.0923	1

**Table 2:** Choice of acid.

	MgO (g)	Borax (g)	Water (mL)	Observation
KH_2_PO_4_ (powder) = 0.816 g	1.132	0.094	1.5	Very resistant
KH_2_PO_4_ crist = 0.837 g	1.049	0.096	1.5	Very resistant
K_2_HPO_4_ = 0.265 g	1.133	0.067	1.4	Very resistant
H_3_PO_4_ = 1 mL	1.032	0.096	2	Very exothermic with gas release and very resistant structure
MgHPO_4_·3H_2_O = 1.073 g	1.042	0.126	2	Very resistant
NH_4_H_2_PO_4_ = 2.8720 ((Mg/P) = 1)	1.078	0.319	2	Very durable with gas release

**Table 3:** Choice of Mg/P ratio.

Mg/P ratio	MgO (g)	KH_2_PO_4_ (g)	Borax (g)	Water (mL)	Observation
Mg/P = 1	1.054	3.036	0.270	1.3	Very resistant
Mg/P = 2	1.062	1.045	0.137	1.2	Very resistant
Mg/P = 3	0.547	0.523	0.059	1	Very resistant
Mg/P = 4	1.066	0.812	0.092	1	Very resistant
Mg/P = 5	0.570	0.370	0.043	1	Resistant
Mg/P = 8	1.023	0.467	0.072	1	Less resistant
Mg/P = 12	1.001	0.270	0.064	1.3	Fragile

**Table 4:** Choice of setting retarders.

	MgO (g)	KH_2_PO_4_ (g)	Water (mL)	Observation
Without retarders	1.001	0.823	2	Quick take
Borax = 0.092 g.	1.066	0.812	1	Moderate hold
Boric acid = 0.096 g.	1.037	0.820	1	Moderate hold
NaCl = 0.093 g.	1.001	0.866	1.3	Quick take

**Table 5:** Different quantities of water.

Water	e/c	MgO (g)	KH_2_PO_4_ (g)	Observation
2 mL	1.089	1.002	0.833	Formation of a homogeneous paste. quick to set and resistant.
4 mL	2.168	1.006	0.839	Weak setting. no dough formation. totally crumbly.
6 mL	3.260	1.006	0.836	Very weak setting. no dough formation. totally crumbly.
8 mL	4.347	1.007	0.837	Very weak setting. no dough formation. totally crumbly.

**Table 6:** Composition of samples for Cs sorption.

	M/P	MgO	KH_2_PO_4_	Borax	Zeolite	Water	pH
S1	2	1.0016	1.6892	0.1340	1.3470	4	9–10
S2	4	1.0054	0.8456	0.0965	0.9232	3.5	7–8

**Table 7:** Parameters of Langmuir, Freundlich, Temkin, and Dubinin–Radushkevich isotherm models.

Model	Sample 1	Sample 2
Langmuir	*b* = 0.025 L/g *q*_0_ = 1.636 mg/g *R_L_* = 0.187 *K_ap_* = 0.144 *R*^2^ = 0.946	*b* = −0.035 L/g *q*_0_ = 4.65 mg/g *R_L_* = 0.042 *K_ap_* = −0.744 *R*^2^ = 0.906
Freundlich	1/*n* = 0.588 *K_f_* = 4.636 *R*^2^ = 0.925	1/*n* = 0.724 *K_f_* = 1.23 *R*^2^ = 0.6513
Temkin	*A* = 1.288 *B* = 16.49 *R*^2^ = 0.903	*A* = 1.31 *B* = 13.8 *R*^2^ = 0.814
Dubinin–Radushkevich	*q_d_* = 39.067 *B* = 0.253 *R*^2^ = 0.591	*q_d_* = 85.66 *B* = 97.89 *R*^2^ = 0.873

**Table 8:** Comparing the present adsorbent to others.

Adsorbent	Capacity (mg/g)	Reference
CHCF-PAN composite	12.5	[27]
Prussian Blue	25.52	[28]
Inorganic adsorbent	27.40	[29]
Conjugate adsorbent	50.23	[29]
AMP-PAN	81.31	[30]
Phosphate-modified Montmorillonite	93.87	[31]
NH4PMA	97.23	[32]
S1	122.108	This study
S2	106.997	This study

## Data Availability

Data will be requested to the authors.
